# Straw Tar Epoxy Resin for Carbon Fiber-Reinforced Plastic: A Review

**DOI:** 10.3390/polym16172433

**Published:** 2024-08-28

**Authors:** Zhanpeng Jiang, Jingyi He, Huijie Li, Yiming Liu, Jiuyin Pang, Chuanpeng Li, Guiquan Jiang

**Affiliations:** Key Laboratory of Wood Materials Science and Engineering, Beihua University, Jilin 132013, China; jzp1578794094@163.com (Z.J.);

**Keywords:** straw tar, Lignin, modification, carbon fiber, epoxy resin, synthesis

## Abstract

The massive consumption of fossil fuels has led to the serious accumulation of carbon dioxide gas in the atmosphere and global warming. Bioconversion technologies that utilize biomass resources to produce chemical products are becoming widely accepted and highly recognized. The world is heavily dependent on petroleum-based products, which may raise serious concerns about future environmental security. Most commercially available epoxy resins (EPs) are synthesized by the condensation of bisphenol A (BPA), which not only affects the human endocrine system and metabolism, but is also costly to produce and environmentally polluting. In some cases, straw tar-based epoxy resins have been recognized as potential alternatives to bisphenol A-based epoxy resins, and are receiving increasing attention due to their important role in overcoming the above problems. Using straw tar and lignin as the main raw materials, phenol derivatives were extracted from the middle tar instead of bisphenol A. Bio-based epoxy resins were prepared by replacing epichlorohydrin with epoxylated lignin to press carbon fiber sheets, which is a kind of bio-based fine chemical product. This paper reviews the research progress of bio-based materials such as lignin modification, straw pyrolysis, lignin epoxidation, phenol derivative extraction, and synthesis of epoxy resin. It improves the performance of carbon fiber-reinforced plastic (CFRP) while taking into account the ecological and environmental protection, so that the epoxy resin is developed in the direction of non-toxic, harmless and high-performance characteristics, and it also provides a new idea for the development of bio-based carbon fibers.

## 1. Introduction

**Figure 1:**
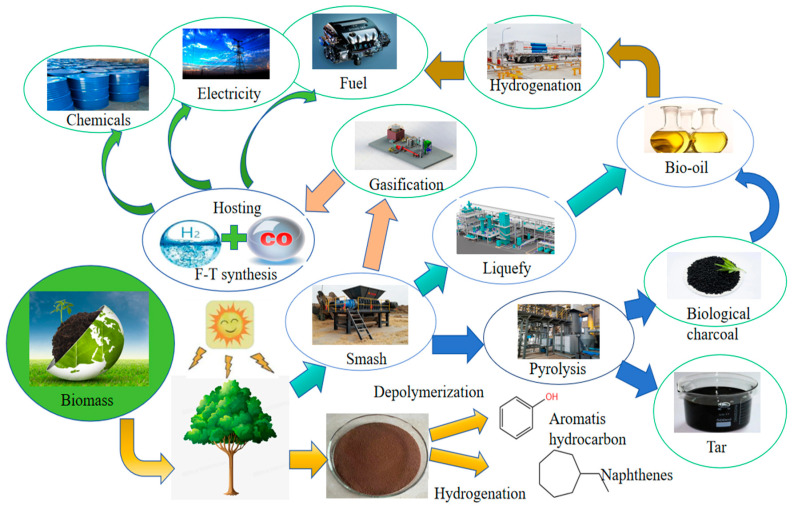
Utilization of biomass resources.

**Figure 2:**
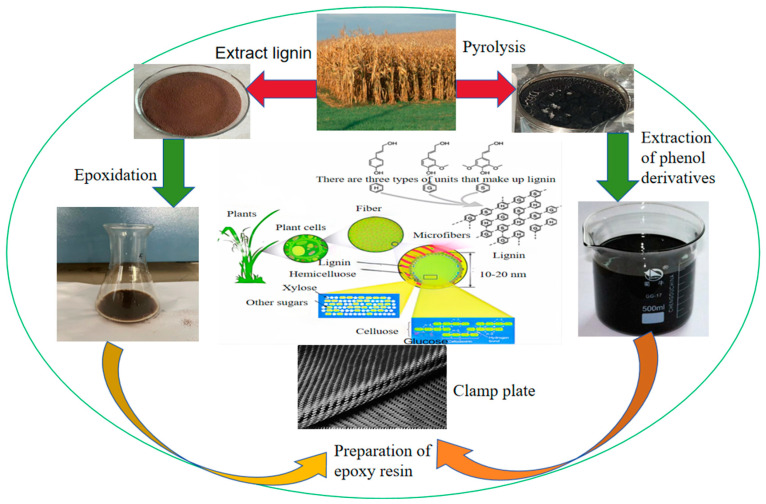
Preparation process.

In 2022, the global demand for carbon fiber was 118,000 tons, compared with 106,900 tons in 2020, a year-on-year growth of 10.4%, with the main application of carbon fiber being for carbon-fiber composite materials in the form of reinforcement materials [1,2,3,4,5,6]. As the “king of new materials” in the 21st century, carbon fiber composites are widely used in aerospace, military, wind power, medical and other high-end industries and play a pivotal role, and the application of carbon fiber-reinforced composites is more in line with the current lightweight, high-intensity development of the concept of various industries [7,8]. Epoxy resin is the matrix of carbon fiber-reinforced plastic (CFRP), and more than 90% of the epoxy resins on the market are realized by the condensation reaction of BPA [9]. However, due to the backward production process and high production cost of BPA epoxy resin products, it is not able to lead the market actively, and there are problems such as greater harm to the environment and human body. Therefore, improving the synthesis process of existing BPA epoxy resins and searching for low-cost, non-polluting biomass substitutes for BPA has become a hot research topic. Exploring low carbon and sustainable energy and material alternatives has become an urgent task. Straw tar has become a non-negligible bio-based feedstock due to its high content of phenolic compounds, and biomass materials are renewable due to their ability to produce fuels, chemicals, and carbon-containing materials, thus making them an excellent choice for replacing fossil fuels [10]. The utilization approach of biomass resources is shown in Figure 1, and the preparation process is shown in Figure 2.

## 2. Corn Stover Tar

### 2.1. Pyrolysis Mechanism

**Figure 3:**
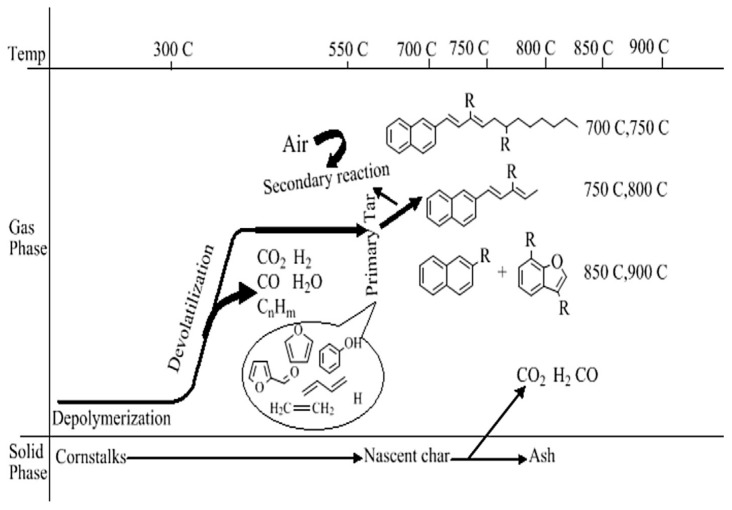
Tar formation process of cornstalk gasification [1].

Corn stover undergoes formation of gaseous products during depolymerization, degradation, and secondary reactions of primary tars, where polycyclic aromatic hydrocarbons (PAHs) with different conjugated bond lengths represent tar compounds of different molecular masses. Lighter-molecular-mass compounds are the main products at higher temperatures. Upon the introduction of air, aromatic compounds with short conjugated bond side chains undergo side chain cracking at high temperatures, resulting in some oxygenated compounds remaining in the corn stover gasification. At the initial stage, different products are produced and the main formation mechanism of gasified tar is related to the formation of PAHs. One of the formation mechanisms of PAHs is considered another formation mechanism of PAHs, i.e., phenols as precursors. Through chloropentadiene production, there is a continuous loss of carbon monoxide and hydrogen radicals from phenols and hydrogen radicals from cyclopentadiene. The two chloropentadienyl groups then combine to form naphthalene. PAHs in gasified tars may come mainly from benzene, such as precursors due to large amounts of lignin. Although corn stover tar may be predominantly phenol-based precursors, corn stover tar may also contain other PAHs, due to the abundance of oxygenated compounds and unsaturated olefins. It can be inferred that hydrogen-rich incorporation chemicals can prevent hydrogen-induced PAHs [11]. The process of corn-stover-gasification tar formation is shown in Figure 3.

Vivanpatarakij et al. [12] developed a method for the complete removal of biotar using a biotar steam reformer, employing co-pyrolysis to regulate the distribution of the products, especially lignocellulosic and carbon materials with a significant synergistic effect to improve the quality of the liquid. Through the distillation process, major components such as aromatic and phenolic compounds can be extracted from the tar, and can be used as intermediates in the manufacture of fragrances, plastics, and dyes, or in hybrid applications such as antioxidants, preservatives, or biomass liquid fuels [13,14].

**Figure 4:**
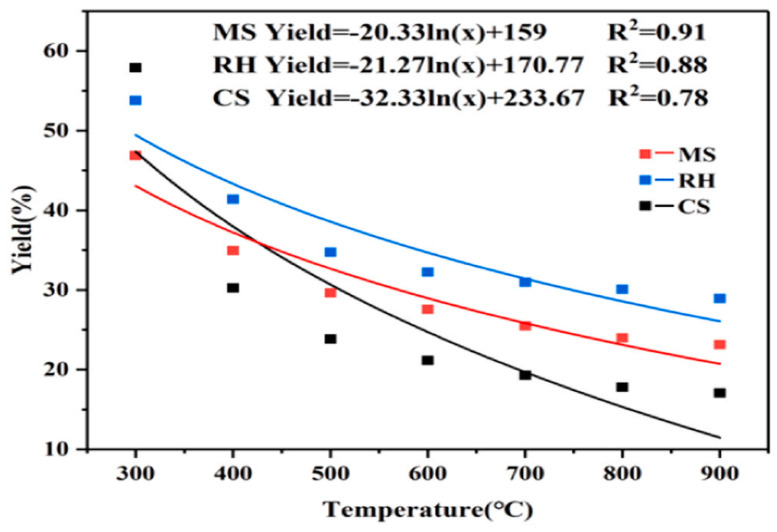
Effects of pyrolysis temperature on the yields of different biochars [15].

Zhang et al. [15] carried out a rapid pyrolysis study of the carbon materials from three materials: corn stover (MS), rice hulls (RH), and cotton stalks (CS) at temperatures ranging from 300 to 900 °C in rapid-pyrolysis experiments to obtain biochar. The results showed that the temperature had the greatest effect on the pyrolysis of these three materials [16]. The oxygenated structures decreased significantly with increasing temperature. In addition, higher pyrolysis temperatures favored the formation of the crystal structure of biochar. In the tar catalytic reforming process, these three representative biochars exhibited excellent catalytic performance, with tar conversion efficiencies of 73.26%, 68.51%, and 71.43% with MS-600 (representing MS biochars generated at 600 °C), RH-600, and CS-600 catalysts, respectively. This study provides systematic support for the high-value utilization of crop-straw biochar. The results of a more detailed study of pyrolysis temperature on the yield of solid products are presented in the figure, and the relationship between them was quantitatively analyzed using fitted curves; the functional relationship established is shown in Figure 4.

**Figure 5:**
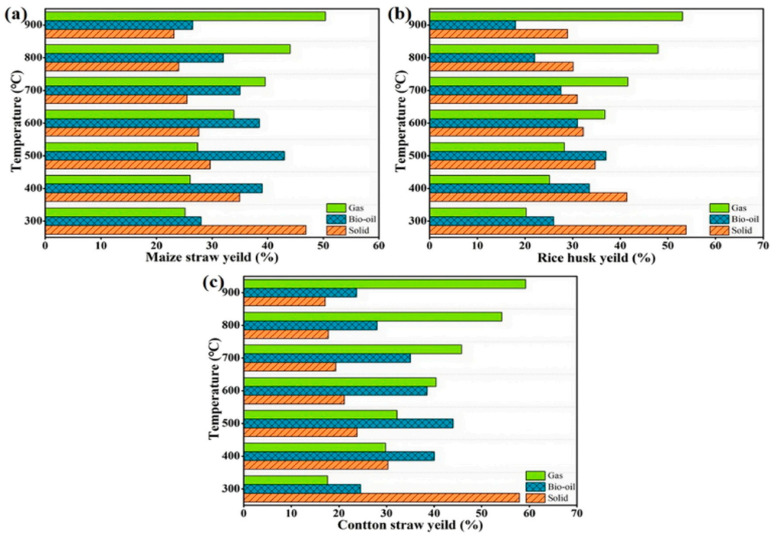
Effect of pyrolysis temperature on product yield: (**a**) MS; (**b**) RH; (**c**) CS [13].

According to the data in Figure 5, the pyrolysis temperature has an important effect on the yield. Firstly, the pyrolysis gas is converted into bio-oil and, finally, biochar is left behind. The yield of biochar was inversely related to the pyrolysis temperature: the higher the temperature, the lower the biochar yield. At 300 °C, the pyrolysis of hemicellulose and some cellulose resulted in the highest biochar yield in cotton stalks [16]. This is due to the biomass material consisting of a large amount of cellulose, hemicellulose and lignin, a hemicellulose decomposition temperature of 200 °C~260 °C, a cellulose decomposition temperature of 240 °C~350 °C, and a lignin decomposition temperature of 280 °C~500 °C, so when the pyrolysis temperature rises to 500 °C, the cellulose, lignin and other components contained in the corn stover are almost all pyrolyzed, resulting in a sharp drop in yield [17,18,19]. Since the lignin content in cotton stalks was higher than the other two agricultural residues, the maximum fluctuation in biochar yield occurred in the range of 300 to 500 °C. In this range, the yield decreased from 57.90% to 23.82%. In the temperature range of 600–900 °C, the biochar yield remained relatively stable [20,21].

### 2.2. Composition

**Figure 6:**
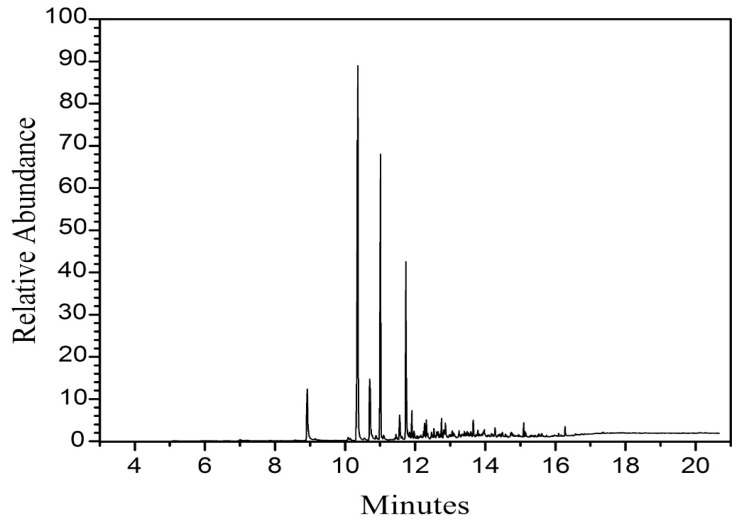
Total ion-flow chromatogram of corn stover tar [22].

Corn stover tar is mainly composed of hydrocarbons, and a small amount contains a small amount of organic matter such as benzene, aldehydes, methanol, phenol, etc. Basically, it does not contain sulfur, which is a serious pollutant of the environment, and it is a more environmentally friendly biomass material than phenol. Similar to the structure of phenol, the synthesis of resin with phenol in straw tar instead of phenol is not only in line with the sustainable development strategy, but can also greatly reduce the cost of phenolic resins, which is a good direction for the future development of the adhesive industry. Chuanpeng et al. [22] used gas chromatography–mass spectrometry (GC-MS) for the synthesis and analysis of corn stover tar. The total ion-flow chromatogram of corn stover tar is shown in Figure 6, and the types of compounds matching the experimental results are shown in Table 1. The composition of corn stover tar is very complex, and many components were detected by GC-MS analysis. However, many of these components are not specific substances. In order to analyze the composition of corn stover tar more accurately, the analysis showed that corn stover tar was mainly composed of phenols, ketones, alkanes, and other substances.

Trans-decahydronaphthalene accounted for the majority, accounting for 34%, and a single substance accounted for more than 50%. Phenolic content is the second, accounting for more than 30%. Given the rich phenol content of corn stover tar, it enables the synthesis of epoxy resins instead of phenol.

**Figure 7:**
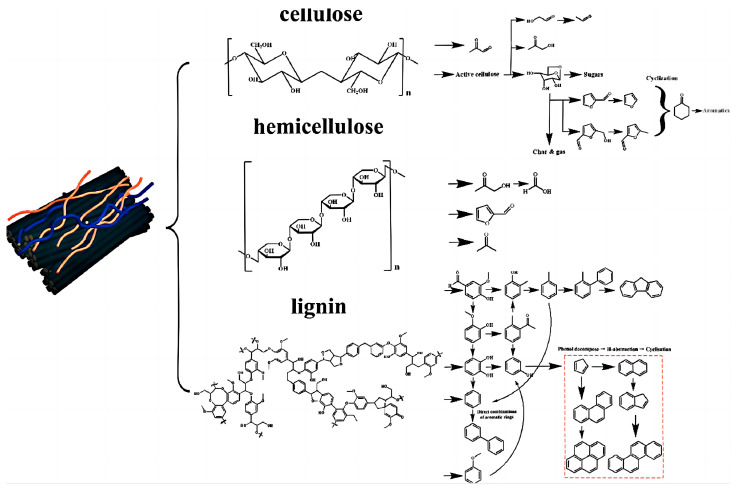
Tar formation pathways derived from the three major biomass components [27].

Biomass is mainly composed of lignin, cellulose and hemicellulose [23]. These three components exhibit different properties during pyrolysis. Hemicellulose decomposes firstly below 225 °C, the main decomposition range of cellulose is 225–400 °C, while the decomposition of lignin obviously occurs at 400 °C. Consequently, biomasses with different proportions of lignin, cellulose and hemicellulose show markedly different activities during gasification. In general, woody crops are rich in lignin, produce more tar and have a slower pyrolysis process. In contrast, herbaceous crops and agricultural wastes are rich in cellulose and hemicellulose, with higher volatile content, fast pyrolysis rates and concentrated pyrolysis intervals [21,24,25,26]. The source pathways of the three main biomass components of tar formation are shown in Figure 7.

### 2.3. Extraction of Phenol Derivatives

With the rapid development of science and technology, the process of extracting phenol derivatives from tar has been continuously improved. Extraction of phenol derivatives is one of the effective ways of realizing the high value-added utilization of straw tar. Understanding the distribution and structural characteristics of phenolic compounds in straw helps to develop efficient separation methods. For the extraction and refinement of crude phenols in straw tar, alkaline extraction, solvent extraction and novel separation methods can be used [28,29].

#### 2.3.1. Extraction Method

Polyphenols are polar, easily destroyed by heat, and structurally unstable. At present, the extraction of polyphenolic components in plants is mainly used in acetone aqueous solution or alcohol aqueous solution at room temperature, in the cold immersion extraction method: in the selection for extraction, glass containers should be chosen rather than metal containers, to prevent polyphenol component and metal ion complexation; for hydrolysis of tannins, extraction should carried out using rapid stirring methods to reduce the occurrence of hydrolysis process: in the process of extraction, many non-polyphenolic impurities will also be extracted, so before and after plant extraction, small polar solvents such as petroleum ether, n-alkane, etc., will be used for degreasing, and then ethyl acetate will be used to extract polyphenols with medium relative molecular mass [30,31].

At room temperature, phenolic compounds are difficult to dissolve in water; as the temperature increases, the solubility of phenolics in water increases, and when the temperature is higher than 200 °C, superheated water vapor can dissolve most of the phenolics in coal tar. Low-order coal tar is rich in phenolic compounds. Phenol extraction with ethanolamine is a promising alternative to the traditional alkali washing method, which causes serious environmental problems The neutral oil content of phenolic compounds extracted by the ethanolamine method with naphthalene compounds predominantly exceeds the metallurgical industry standard of our country, which restricts the industrial application of phenol extraction by the ethanolamine method [32]. Here, a conceptual process involving ethanolamine extraction, azeotropic distillation and crystallization is proposed to reduce the neutral oil content. The naphthalene compounds were efficiently separated from the phenolic compounds as ethanolamine and naphthalene compounds formed a minimum azeotrope. Thereafter, the naphthalene compounds were separated from the ethanolamine by crystallization. Finally, both naphthalene compounds and phenolic compounds were obtained as products. The separation characteristics of the new process were comprehensively analyzed, and the key process parameters were optimized by experiments and simulations. The process can not only overcome the secondary pollution caused by the alkali washing method, but also overcome the problem of high neutral oil content in the traditional organic solvent extraction method [33,34,35].

Rajapaksha et al. [36] extracted polyphenols from spent black tea using a pilot scale process for semi-continuous subcritical solvent extraction (SSE) of polyphenols from SBT. Treatment of SBT with ethanol–water (50% *w*/*w*) as a solvent under certain conditions significantly increased the yield of polyphenols with antioxidant activity, compared to hot water extraction (HWE). SSE increased the content of soluble substances in the extracts, compared to HWE. Minowa et al. [37] used methanol-mediated extraction with an application to wood-carbonization tar. Phenolic compounds can be extracted from wood tar, which indicates the possibility of selective extraction of phenolic compounds, although wood tar consists of a wide range of oxygenated compounds such as alcohols, phenols, ketones, esters, ethers, aldehydes, carboxylic acids, furans, etc., and this extraction technique is expected to be a new method for wood tar utilization. Rodriguez-Seoane et al. [38] utilized subcritical water extraction and supercritical CO_2_ extraction using green technology, which has a shorter extraction time than conventional solvents under atmospheric-pressure oscillation conditions. Subcritical water extraction was carried out at temperatures ranging from 140 to 240 °C and supercritical CO_2_ extraction at different pressure temperatures and ethanol concentrations. Subcritical water extraction in non-isothermal operation during heating to 160 °C provides up to 30% extraction.

#### 2.3.2. Lye Extraction Method

The alkali extraction method mainly uses the phenolic hydroxyl group in phenolic compounds, which is weakly acidic, and in alkali neutralization reaction, the phenolic compounds are changed from the molecular state into the ionic state of the phenol salt, so as to achieve the purpose of separation. Phenolic compounds are extracted with different alkali solutions and reacted with sodium hydroxide to obtain water-soluble phenolic sodium salt, so that phenols are transferred to aqueous solution in the form of phenolic sodium salt, and then acidic substances such as sulfuric acid or carbon dioxide are used to reduce the phenolic sodium salt to phenolic substances, so that phenols are enriched. Since the acidity coefficient of some phenolic compounds is close to that of carbonic acid, it is difficult to realize effective separation. At present, sodium hydroxide-solution extraction is the only phenol extraction method that realizes an industrial application, and has the characteristics of simple process, mature technology and high extraction rate of low-grade phenols, but the separation process will produce a large amount of waste acid and alkali, which easily causes equipment corrosion and environmental pollution.

Borrega et al. [39] extracted phenolic compounds from industrial spruce bark with sodium hydroxide and showed that 20–27% of the bark could be extracted as polyphenols, mainly tannins and some lignin. Polyphenols extracted at 100 °C, 15% sodium hydroxide and 160 °C, 24% sodium hydroxide were recovered by acid precipitation and tested as surfactants. Despite the different compositions, the polyphenol-rich precipitates had similar surface activity and were soluble under neutral conditions. In addition, more than 80% sugar yield was obtained by enzymatic saccharification of the extracted bark residues. These results suggest that alkali extraction is a promising extraction technique.

At present, NaOH solution extraction is the only phenol extraction method that realizes an industrial application, which is characterized by a simple process, mature technology, and high extraction rate of low-grade phenols, but the separation process generates a large amount of waste acid and alkali, which is prone to cause equipment corrosion and environmental pollution [40]. The development trend of this field is discussed; the analysis and identification of phenols need to be combined with a variety of advanced means, and a multi-level, all-round analysis of its composition and structure. The extraction of crude phenol can speed up the selection of environmentally friendly and efficient extractants to promote the progress of the application of the new extraction method and the crude phenol refining, in the focus on the development of meso- and p-cresol and mixed dimethylphenol high-efficiency purification process at the same time, to strengthen the research efforts of the extraction of high-level phenol [41].

### 2.4. Utilization of Lignin

Lignin is a natural polymer compound with a complex phenolic structure; its structure is mainly composed of phenylpropane-structure monomers through free radical coupling to intermolecular connected carbon–oxygen bonds and carbon–carbon bonds. The composition of multiple phenylpropane basic structures makes the basic structure of lignin very similar to that of bisphenol A. Moreover, lignin has the great advantage of renewability and economy.

### 2.5. Lignin Structure and Its Component Composition

**Figure 8:**
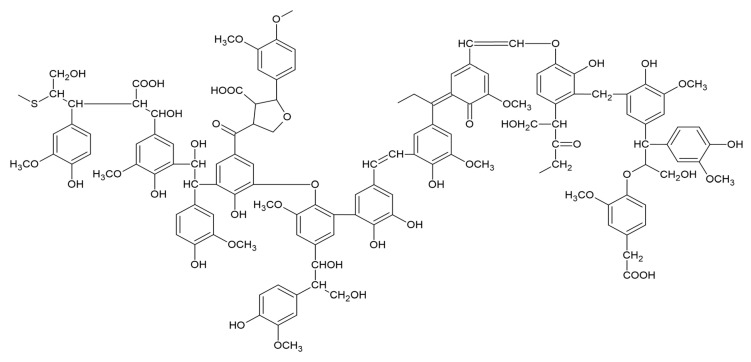
A structural formula of lignin (Huang et al., 2019) [40].

Natural lignin is mainly composed of three elements: carbon, hydrogen and oxygen, and will contain a small amount of nitrogen in some graminoid lignin, while the elemental composition of lignin will be different with the change in separation method. Lignin has the highest content of natural macromolecular aromatic polymers in the biological world; its structure is composed of phenylpropane (C6-C3), constituting the main skeleton, and phenylpropane structural units are dehydrogenated and then connected in disorder through the chemical bonding, which leads to the formation of a three-dimensional mesh structure with a large number of benzene rings of aromatic compounds. Natural lignin in nature is mainly composed of Sinapyl alcohol, Coniferyl alcohol and p-Coumaryl alcohol, and is composed of three monomers, namely, S-type lilac propane, G-type guaiacol propane and H-type p-hydroxyphenyl propane. Lignin is a constituent of plant cell walls, in addition to cellulose and hemicellulose [42]. The deduced structural formula of lignin is shown in Figure 8.

### 2.6. Modification of Lignin

Lignin is a very complex natural polymer; the relative molecular mass ranges from several hundred thousand to several million, resulting in it being more difficult to deal with. It must be degraded to be utilized; the structure of lignin contains methoxy, phenolic hydroxyl, carboxyl and carboxymethyl, and many other functional groups and chemical bonds participating in the chemical reaction. There is a greater potential for providing the possibility of its chemical modification to achieve the comprehensive utilization of the possibilities [43].

#### 2.6.1. Hydroxymethylation Modification

Lignin has a large relative molecular mass, a large spatial site resistance of the active site on the aromatic ring, and low reactivity. Lignin itself will constantly condense, and one of the ways to improve the reactivity of lignin is to introduce reactive groups, which can increase its activity without significantly increasing the molecular weight of lignin. Formaldehyde is a good choice. The hydroxymethylation modification of lignin not only improves its hydroxyl content, but also reduces the relative molecular mass of lignin; hydroxymethylation modification of lignin is important for the preparation of nanoparticles and epoxy resin research [44].

**Figure 9:**
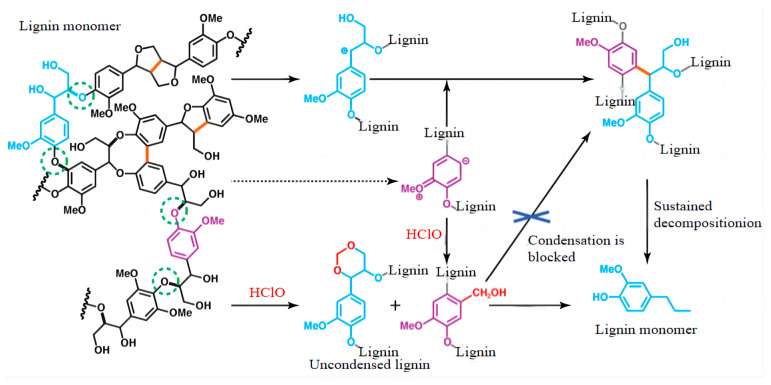
Synthesis mechanism of lignin hydroxymethylation [46].

The introduction of hydroxymethyl groups at the C5 position of the G-unit in the lignin structure results in further crosslinking condensation reactions between the lignin macromolecules and reduces the spatial site resistance of the reactive functional groups. Under alkaline conditions, aldehydes can be two-molecule interactions, where the α-hydrogen atom of one aldehyde is added to the carbonyl oxygen atom of the other aldehyde, and the rest of the aldehydes are added to the carbonyl. The enhancement of lignin’s reactivity requires the increase of the hydroxyl content in lignin, while controlling the molecular weight of lignin [45]. The lignin hydroxymethylation reaction mechanism is shown in Figure 9.

Aini et al. [47] dissolved lignin in distilled water and then added formaldehyde for further reaction, which ultimately leads to hydroxymethylated modified lignin. With a molar mass of 4732 g/mol, the composites synthesized by its epoxidation showed higher compatibility and interfacial adhesion than that of the control group with unmodified lignin, and the curing rate, crosslinking density, and flexural strength were significantly improved, as well as the mechanical properties, such as tensile properties and hardness.

#### 2.6.2. Amine Modification

**Figure 10:**
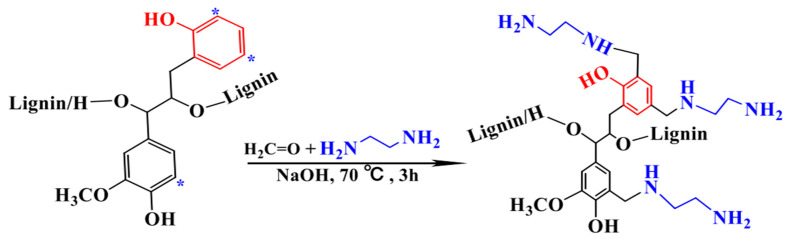
Diagram of lignin amination modification mechanism [51].

The mechanism is shown in Figure 10. Lignin amination modification is similar to hydroxymethylation; the same as in the lignin benzene-ring active site to introduce groups, the lignin structure of the phenolic hydroxyl group in the neighboring position can be reacted with amines and aldehydes, and the reaction process of active hydrogen atoms are replaced by aminomethyl, so that the product’s nitrogen content will be increased, through the free radical-type grafting reaction to introduce the active primary, secondary, or tertiary amine groups in its macromolecular structure, which are based on ether bonding grafted onto the lignin molecule [48]. The activity of lignin was improved by amination modification. When lignin undergoes the Mannich reaction, the hydrogen atoms in the neighboring and para positions of the phenolic hydroxyl group on the benzene ring and the neighboring position of the carbonyl group on the side chain are more active, and can be easily reacted with aldehydes and amines, thus generating lignin amine [49,50].

**Figure 11:**
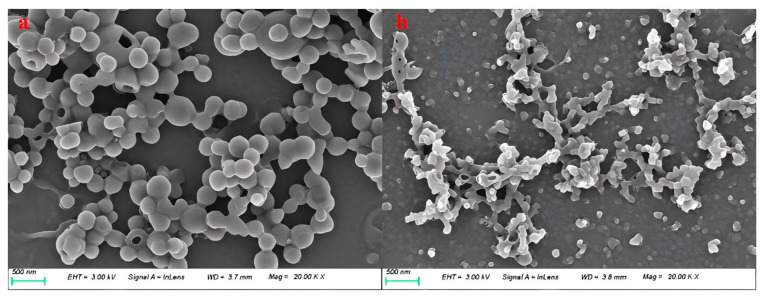
The morphologies of lignin (**a**) and aminated lignin (**b**) [52].

The unmodified alkali lignin and amino lignin are shown in Figure 11. It is spherical in shape and has a more pronounced agglomeration phenomenon, with a particle size of about 250 nm, as shown in Figure 11a. The two figures are compared: aminated lignin presents small particles, with a particle size less than 100 nm, and it is obvious to see that there are no agglomerations, as shown in Figure 11b.

**Figure 12:**
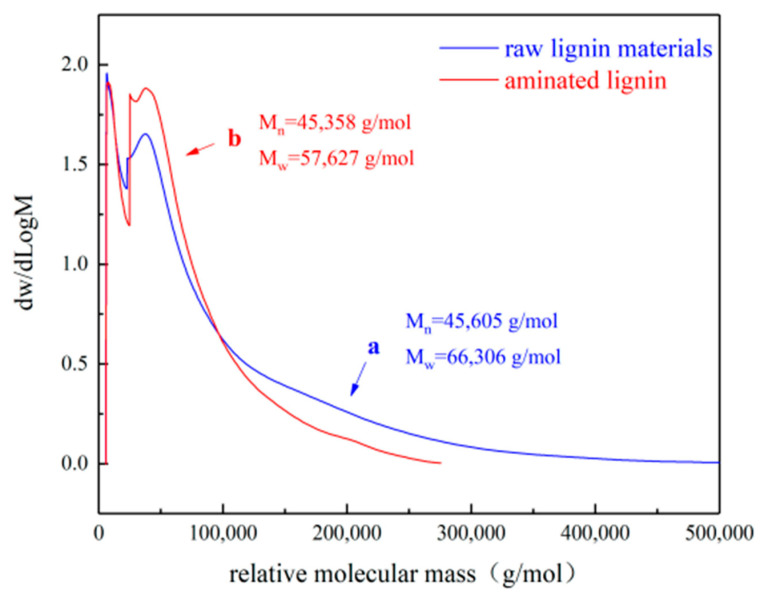
Molecular weight distribution of the generated aminated lignin and the lignin feedstock used [52].

The content of small- and medium-molecular-weight substances in lignin did not change significantly after the ammonification reaction. And the content of large-molecular-weight substances between 40,000 and 100,000 g/mol increased significantly. Lignin was degraded during the ammonification reaction, resulting in lower-molecular-weight aminated lignin. The number for the average molecular weight of the lignin feedstock was 45,605 g/mol, while the average molecular weight was 66,306 g/mol. After reflection, the molecular weight distribution was quite wide, as it contained a large amount of large-molecular lignin with a molecular weight of more than 300,000 g/mol. The number for the average molecular weight of the aminated lignin was 45,358 g/mol, while the average molecular weight was 57,627 g/mol. There is little difference in the average molecular weight after amination, and the average molecular weight drops considerably. The root cause is the alkali hydrolysis reaction of lignin during amination, which leads to the hydrolysis of high molecular weights, thus lowering the average molecular weight. The molecular weight distribution of the resulting aminated lignin and the lignin feedstock used is shown in Figure 12.

Mendis et al. [53] formed several types of lignin–epoxy composites by dissolving lignin in aliphatic amines, where the lignin–amine solution was modified by hydration and the Mannich reaction, and epoxy resin was synthesized by dry epoxidation. The resulting composites exhibited a two-phase microstructure of lignin-rich epimers, and the aminated lignin samples showed glass transition and mechanical properties similar to those of the pure-epoxy samples, with improved modulus of elasticity and fracture strength. Pan et al. [54] investigated aminated lignin containing a large amount of reactive amines as a curing agent to be directly mixed with epoxy resins. The results of thermogravimetric and dynamic thermodynamic analyses showed that the aminated lignin-cured epoxy resin had better thermal stability compared with the epoxy resin cured with ordinary curing agents; and the mass loss of the epoxy resin cured by aminated lignin before 300 °C was only about 2.5%, the glass transition temperature was increased from 79 °C to 93 °C, and the heat distortion temperature from 70 °C to 84 °C. Xin et al. [55] developed a novel two-step method to prepare Burl lignin amines. This further converts oxidized lignin to lignin amine via reductive amination. Ammonia was used in the second step to obtain highly reactive primary lignin amines. Oxidation and reduction showed relatively high yields of 80.0% and 91.2%, respectively. For comparison, the lignin was partially depolymerized by mild hydrogenolysis and then the partially depolymerized lignin was also converted to lignin amine, using the same method [56,57,58,59,60,61,62].

## 3. Lignin Nanoparticles

As the large molecular weight as well as insolubility of traditional lignin limits effective utilization, the conversion of lignin into nanoparticles overcomes the inherent drawbacks of lignin such as multiphase, poor dispersion, and oversized particles, and provides a completely new route for the preparation of lignin-based functional materials [63,64,65,66].Lignin nanoparticles are widely used in functional epoxy resins, nano adhesives, and other fields, due to their excellent properties such as renewability, degradability, and surface activity. Meanwhile, lignin nanoparticles can be compounded with different kinds of nanomaterials, which greatly improves the added value of their applications, and at the same time, facilitates environmental protection and high-value utilization of biological resources [67,68,69,70,71].

**Figure 13:**
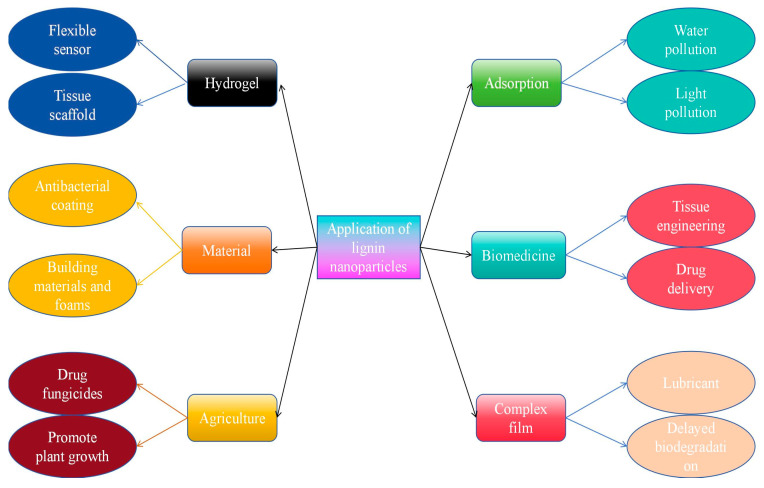
Application of lignin nanoparticles in various fields in recent years.

When the small particle size enters the nanoscale, it itself has a quantum size effect, small size effect, surface effect and macroscopic quantum-tunneling effect, and thus exhibits many unique properties, such as better miscibility with polymers, large specific surface area, improved chemical reaction properties, lower melting point, changes in mechanical properties, changes in magnetic properties and changes in optical properties. The heterogeneity of lignin, including its complex chemical structure and wide molecular-weight distribution, results in uneven and modest properties, thus severely limiting its value-added applications. Nanosizing lignin helps to increase intermolecular collisions, allowing it to penetrate inside the reaction. The pathways for the utilization of nanolignin are shown in Figure 13.

### 3.1. Antisolvent Precipitation Method

The antisolvent method is considered to be the “standard method” for the preparation of lignin nanoparticles (LNPs). This method was originally proposed by Frangville et al. [72], who developed two different methods for dissolving alkali lignin in ethanol and solution, respectively, and then precipitating it with hydrochloric acid solution The researchers used different sources of lignin, employed different solvents and anti-solvents, and demonstrated the application of LNPs in several fields. For example, Yearla and Padasree [73] used acetone solution (9:1, *v*/*v*) as a solvent and meta-ionized water as an anti-solvent to facilitate the precipitation of LNPs. Due to the low solubility of lignin in water, most researchers have used water as an antisolvent, and, in addition, supercritical carbon dioxide has also been used as an antisolvent. Most of the LNPs prepared using the antisolvent method have a well-defined shape and some stability. Since the preparation of LNPs by the antisolvent method inevitably requires the use of some organic reagents or acids and bases, it results in an increase in cost and is also harmful to the environment. In order to overcome these disadvantages and problems to the maximum extent, recycling and reusing the solvents is a good solution.

**Figure 14:**
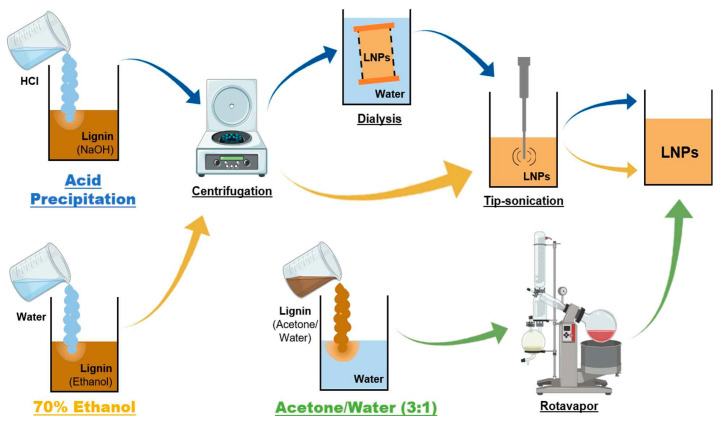
Overview of the different approaches for the LNP preparation used in this study: acid precipitation (blue arrows), 70% ethanol (yellow arrows), and acetone/water 3:1 (green arrows) [74].

Figueiredo et al. [74] successfully prepared homogeneous nanoparticles using three environmentally friendly solvent-resistant precipitation methods that depend on the lignin source and its extraction process, which can influence the composition and properties of the original lignin, such as solubility and phenolic hydroxyl groups. We used three different technological lignins as starting polymers for the preparation of LNPs: softwood sulfate lignin promoter (LB), wheatgrass/sakandha grass alkaloid proteins 1000 (PB), and hardwood BLN birch lignin (BB) for the preparation of LB-LNPs, PB-LNPs, and BB-LNPs, respectively. Three environmentally friendly anti-solvent precipitation methods were employed, with 70% ethanol, acetone/water (3:1), and aqueous sodium hydroxide solution. For the acid precipitation method, we optimized the precipitation conditions using different acids (nitric, sulfuric, and hydrochloric), and hydrochloric acid was chosen because it provided the highest yield of LNPs in acid. The three protolignans were dissolved in aqueous sodium hydroxide solution, precipitated with aqueous hydrochloric acid solution, further centrifuged, dialyzed to remove the salt, and ultrasonicated to re-disperse the LNPs. The results showed that the acetone/water (3:1) method is the most efficient way to obtain such lignin nanoparticles, and can greatly increase their application in many fields. The reaction flow is shown in Figure 14.

### 3.2. Self-Assembly Method

**Figure 15:**
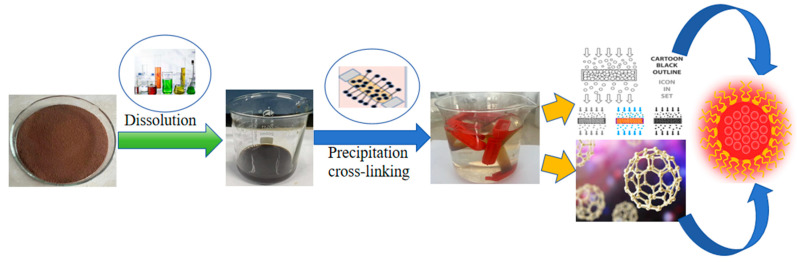
Process for preparing nanoparticles by self-assembly method.

The self-assembly method for the preparation of lignin nanoparticles relies on the amphiphilicity of lignin molecules, which self-assemble into spherical particles under certain conditions. The self-assembly process is not a simple superposition of weak forces among a large number of atoms, ions, and molecules, but rather a number of individuals spontaneously associated and assembled together to form a tight and orderly whole. The lignin was dissolved and insoluble impurities were removed from the pretreated alkaline lignin. The pH of the solution was adjusted to a lower level by adding acid, and the solution was dialyzed with a dialysis bag to allow the lignin to precipitate and crosslink, and finally ultrasonicated to form homogeneous and stable lignin nanoparticles; the reaction is shown in Figure 15.

Zhao et al. [75] prepared lignin nanoparticles (LNPs) by the self-assembly method in modified alcohol solution. The nanoparticles prepared by this method were examined by SEM and DLS, and the homogeneous, stable, and uniformly dispersed lignin nanoparticles were successfully prepared. The epoxy resins prepared from the lignin nanoparticles showed excellent properties and broad application prospects. Almeida et al. [76] investigated the process of lignin macromolecules’ self-assembling into lignin nanoparticles (LNPs) in green solvents and through the detailed study of different variables in detail, to provide clues for the customized production of LNPs. In lignin solvents, ethanol allowed the preparation of LNPs with the lowest hydrodynamic diameter (Method B = 127.4 nm), while the largest particles were obtained with ethylene glycol (Method A = 7820 nm). These latest particles were characterized as heterogeneous, irregular and highly aggregated compared to their γ-pentolactone counterparts, which showed the most homogeneous (PDI = 0.057–0.077) and spherical particles. In addition, reduced lignin solution loading decreases LNP size and zeta potential. Dialysis of the samples resulted in the formation of LNPs with lower hydrodynamic size, reduced aggregation, and higher homogeneity. In addition, dialysis provided high stability to the LNPs and avoided particle agglomeration.

## 4. Lignin Epoxidation

Epoxy resin is a class of organic polymers containing more than two epoxy groups in the molecular structure, which can be crosslinked with many types of curing agents to form insoluble, non-fusible polymers with a three-way mesh structure, with excellent insulating properties, mechanical properties, and chemical stability, etc. [76,77,78,79,80,81]. It is widely used in adhesives, coatings and other fields. Hydrogen peroxide is known as a green oxidizing agent, which combines with lignin to form epoxy lignin to prepare bio-based epoxy resins. The traditional way of synthesizing epoxy resins in the market is the condensation of bisphenol A with epichlorohydrin, which is not only costly but also toxic and harmful, and the replacement of epichlorohydrin with bio-based epoxy lignin has a good application prospect [82,83,84,85,86].

**Figure 16:**
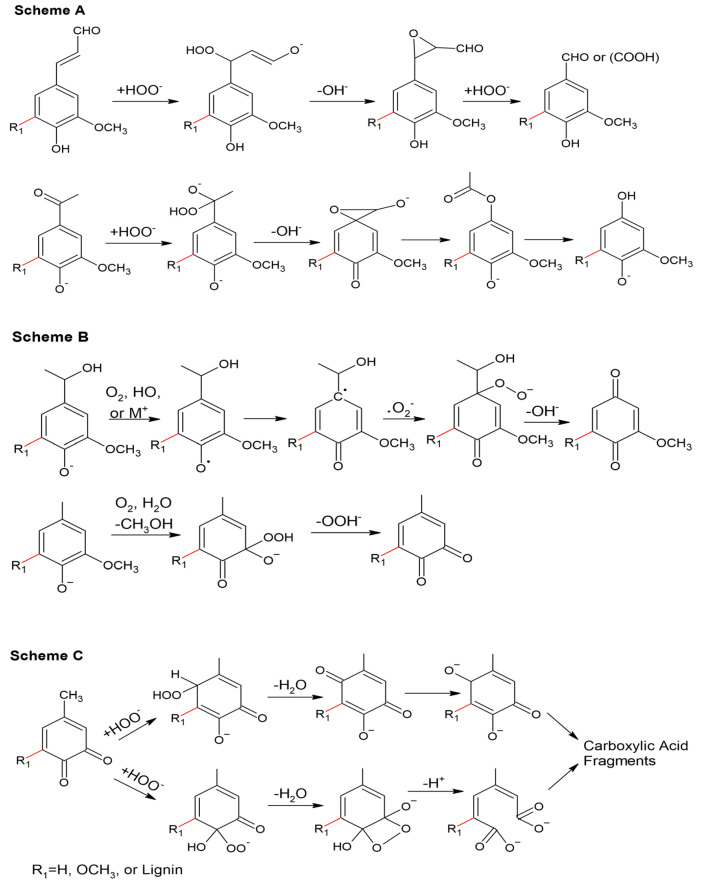
Hydrogen peroxide oxidation of phenolic-lignin mechanisms: (**Scheme A**) conjugated side-chain oxidation, (**Scheme B**) aromatic ring oxidation to benzoquinone structures, (**Scheme C**) aromatic ring cleavage [40].

The phenolic structure of lignin is easily cleaved by alkaline hydrogen peroxide to produce the corresponding aromatic aldehyde, and the reactant hydrogen peroxide nucleophilically attacks the Cα position to form an epoxide. There is quantitative cleavage of the α-carbonyl structure in the phenol unit. In the presence of the phenolic unit, the reaction proceeds via a Dakin reaction and intermediate formation of the epoxide, which is rapidly hydrolyzed under alkaline conditions, accompanied by the cleavage of Cα-C1 to form the corresponding methoxyhydroquinone derivative. The mechanism representing this cleavage reaction is shown in Figure 16, Scheme A [87].

For the α-carbinol structure, the reaction proceeds through the formation of a quinone methyl intermediate, which then proceeds as shown in Figure 16, Scheme B. When the quinone methyl group reacts rapidly with superoxide ions, the corresponding hydrogen peroxide is formed. The hydrogen peroxide derivative then forms a p-quinone-type structure. In the absence of a reactive part at the α position, the nucleophilic attack of hydrogen peroxide occurs in the ortho position, where the methyl group is eliminated. Subsequently, o-quinone-type structures are then formed [88]. It is feasible to have multiple reaction sites in the o-quinone and p-quinone rings, which are attacked by hydrogen peroxide anions resulting in ring-opening products, as shown in Figure 16, Scheme C.

**Figure 17:**
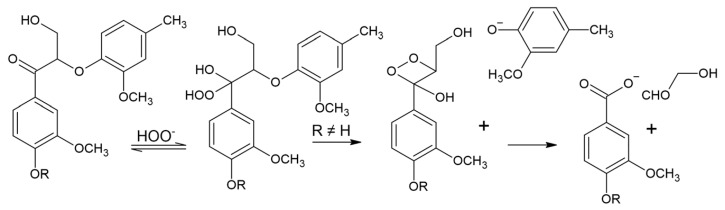
Hydrogen peroxide oxidation of non-phenolic lignin with aryl-α-carbonyl conjugated structures (Kadla and Chang, 2001) [90].

Unlike the epoxidation of lignin, hydrogen peroxide can react with non-phenolic lignin structures. In nonphenolic units with an aryl-α-carbonyl conjugated structure, intramolecular nucleophilic attack results in the formation of phenolic hydroxyls and dioxane intermediates, decomposition to benzaldehyde, and, finally, oxidation by benzaldehyde. In this case, the Dakin reaction is ruled out due to the absence of a free phenolic hydroxyl group, as shown in Figure 17 [89].

### 4.1. Lignin and Its Derivatives’ Epoxidation Synthesis of Epoxy Resin

The direct epoxidation of lignin refers to the direct epoxidation of lignin and its related derivatives in an alkaline environment without modification, by directly epoxidizing the phenolic hydroxyl group and alcohol hydroxyl group contained in lignin under the action of a catalyst to generate epoxy resin, so as to achieve the purpose of synthesizing epoxy resin with lignin instead of petroleum-based raw material bisphenol A. Epoxy lignin, instead of the industrial product epichlorohydrin, is a green method that reacts with phenol derivatives, which provide hydroxyl groups. Finally, a bio-based epoxy resin is obtained.

Jung et al. [91] extracted methanol-soluble lignin sulfate (MSKL) by methanol graded separation for lignin epoxidation, and directly epoxidized it to lignin-derived epoxy resins by a two-step epoxidation reaction. The presence of epoxy groups in the lignin-derived epoxy resin was confirmed by studying infrared spectroscopy (FT-IR), hydrogen nuclear magnetic resonance spectroscopy (1H-NMR), and thermogravimetric analysis (TGA), and its heat-resistant properties were comparable to those of commercially available epoxy resins, suggesting that lignin has the potential to be used as a replacement for bisphenol A in the epoxy resin industry. Asada et al. [92] used tetramethylammonium chloride solution as a catalyst in an alkaline environment, and heated the suspension to 70 °C in a nitrogen environment to react, so that methyl ethyl ketone was used as a solvent to close the loop, and lignin epoxy resin was obtained. After analysis and testing, it can be found that the yield of lignin-derived epoxy resin is very similar (63.4~68.2%) to that of bisphenol A (70%), and that the thermal stability of cured lignin-epoxy resin is excellent. This indicates that lignin-based epoxy resin can be synthesized using the same synthetic route as bisphenol A-based epoxy resin; lignin derived from biomass is expected to be a candidate to replace bisphenol A-derived petroleum-based epoxy resin, and it can be used in the production of composite materials. It not only improves the degradability of existing composite products, but also greatly improves its physical properties.

### 4.2. Direct Blending of Lignin and Its Derivatives with Epoxy Resins

Lignin is the only renewable resource with aromatic structure discovered so far, so it is considered to be an excellent candidate for synthesizing bio-based polymers. At the end of the 19th century, foreign researchers began to study and explore the direct value-added characteristics of lignin The most direct and effective method is to blend lignin with epoxy resins, and to utilize the reactive groups in lignin to improve the properties of epoxy resins [93].

Nonaka et al. [94] used industrial sulfated lignin with 2.70% phenolic hydroxyl groups as the sample, and the water-soluble epoxy compounds used polyethylene glycol diglycidyl ether and diglycidyl ether of emulsion-type bisphenol A to blend with the lignin sample. The industrial sulfated lignin was blended with the epoxy compounds in an alkaline environment. The curing gelation rate of the blended systems with different lignin contents varied significantly, and the rate of change of the peak temperature of the modulus of dissipation was faster for different lignin contents. The relationship between the peak loss-modulus temperature and its glass transition temperature is roughly compatible. The cured resins were shown to have excellent compatibility and good dynamic mechanical properties, by dynamic mechanical measurements.

## 5. Reaction of Epoxy Lignin with Phenol Derivatives

Epoxy resins and their cured substances are meant to be cured substances with a crosslinked structure after the reaction of epoxy groups containing more than two epoxy groups (Epoxy Group) with multifunctional compounds. They are characterized by high reactivity and wide range of applications, and the molecular chain grows after ring-opening polymerization or an addition reaction with other compounds [95].

Epoxy lignin is used instead of epichlorohydrin, and phenol derivatives instead of bisphenol A. After epoxidation of lignin, etherification with phenol derivatives occurs to form polymers, and the product contains ether bonds and hydroxyl and epoxy groups, which allows the epoxy resins to form a better crosslinking network. The hydrogen atoms of hydroxyl groups in the phenol derivatives in the straw are etherified by the aryl groups in the epoxy lignin, and the two alcohols or phenol hydroxyl groups are between them. Through condensation reactions between alcohols and alcohols, alcohols and ethers, alcohols and haloalkanes, and other compounds, the alcohol molecule first loses a proton to form an alcohol negative ion. The alcohol negative ion then undergoes nucleophilic attack with another alcohol molecule or another reactant to form an ether compound.

The presence of the ternary-ring height tension of the epoxy group in epoxy lignin allows the epoxy group to undergo a ring-opening reaction with a nucleophilic reagent, such as a hydroxyl group, under certain conditions, to form an ether bond. The presence of the unique spatial site resistance of lignin makes the reaction difficult and requires the use of strong catalysts with better catalytic effect, such as barium hydroxide, potassium hydroxide, etc., which serve to accelerate the rate of proton transfer and nucleophilic attack. In the formation of reaction intermediates, unstable intermediates such as ether anions or ether cations may be formed between the alcohol anion and the reactants of the nucleophilic attack. These intermediates act as catalysts during the reaction and facilitate the reaction [96].

## 6. Epoxy-Resin Curing Agent

**Figure 18:**
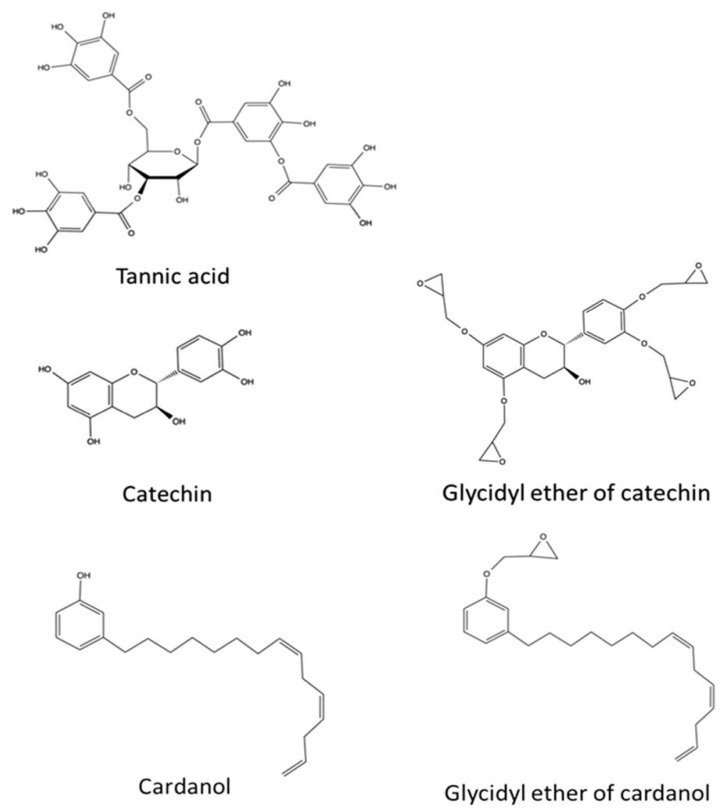
Structures of tannic acid and catechin, two main components of tannins. Also pictured is cardanol, which is a derivative of cashew nut-shell liquid. Glycidyl ether of catechin and cardanol with their epoxy group functionalities are also shown [97].

The synthesis of epoxy resins into crosslinked polymers with excellent mechanical and thermal properties requires the addition of curing agents for curing. These curing agents can be a variety of compounds, such as amine curing agents, anhydride curing agents, or Lewis acids or bases. The properties of the material depend critically on the type of epoxy resin and the type of curing agent used. Bisphenol A makes up the majority of the market for epoxy additives; however, BPA is banned in many countries due to its high toxicity, which creates additional environmental and health concerns. Curing agents are epoxy resins which provide high adhesion and excellent chemical and thermal resistance, and bio-based curing agents have become a hot topic of research. A study by Baroncini et al. [97] compared the polymer formation of epoxy catechins, green tea extracts and BPA-based resin systems. Compared to the BPA-based system, the green tea extract contained a variety of catechin-based phenolic compounds, and the catechin-and-green-tea extract-based resins exhibited higher storage moduli of 2340 MPa and 1290 MPa, at 30 °C, indicating improved mechanical properties. This suggests that cured resins based on epoxidized green tea extract can create viable bio-based epoxy resins. The figure also shows the structures of tannins and catechins, which are the two main components of tannins. In addition, the figure shows cashew nut phenol, which is a derivative of cashew nutshell liquid, and the glycidyl ethers of catechins and cashew nut phenol and their epoxy group functionalities are shown in Figure 18.

## 7. Carbon-Fiber-Surface Treatment

The sizing agent contains a solution or emulsion of polymer components, which can be impregnated, sprayed, etc. to coat the surface of carbon fiber (CF), forming a polymer film to improve the wettability of CF (carbon fiber), and can act as a coupling agent, thus improving the interfacial bonding between the fiber and the resin. This method is simple, easy to implement, and effective, and is a commonly used treatment in industry [98,99,100]. However, ordinary sizing agents have certain defects in temperature resistance, and for carbon fibers, their smooth surface, low surface energy and lack of active groups show physical and chemical inertness, resulting in weak interfacial bonding between fibers and resin, making it difficult to give full play to the performance advantages of carbon fibers. Therefore, surface modification of carbon fibers to improve the interfacial bonding properties of fibers and resins is an important way to advance the application of composites [101]. So far, various methods have been applied to the surface modification of carbon fibers. Oxidation of the CF surface using oxidizing gases at high temperatures produces both reactive groups and etching on the fiber surface. This method is easy to operate, has a low cost, and is suitable for mass-production applications, but its treatment conditions are more difficult to control, and it is easy to cause fiber damage, thus affecting the mechanical properties of the products [102,103,104,105].

**Figure 19:**
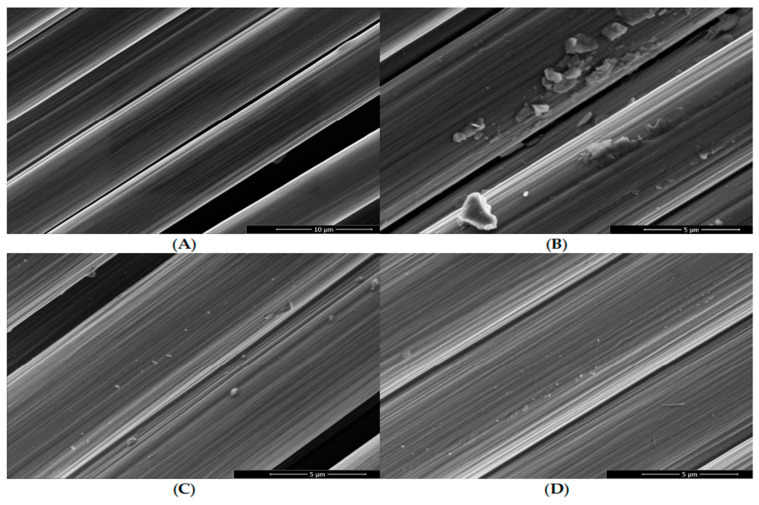
Scanning electron micrographs taken from the untreated reference fiber sample (**A**) and differently oxy-fluorinated carbon fiber samples: 1 vol% fluorine (**B**), 3 vol% fluorine (**C**), 5 vol% fluorine (**D**) [106].

The following figure shows the modification treatment of carbon fibers with different concentrations of fluorine; it can be seen from Figure 19B–D that by increasing the fluorine content in the reaction chamber, the streaks caused by fiber stretching are shown more clearly. This is related to the roughening of the surface structure of the fibers, where all modified fibers have porous regions along their axes and their surfaces exhibit particle contamination. Comparing the modified fibers with the untreated fiber samples (Figure 19A), a reduction in fibers was observed. The fibers were completely stripped when treated with a high concentration of fluorine (10 vol% fluorine) in the process gas.

**Figure 20:**
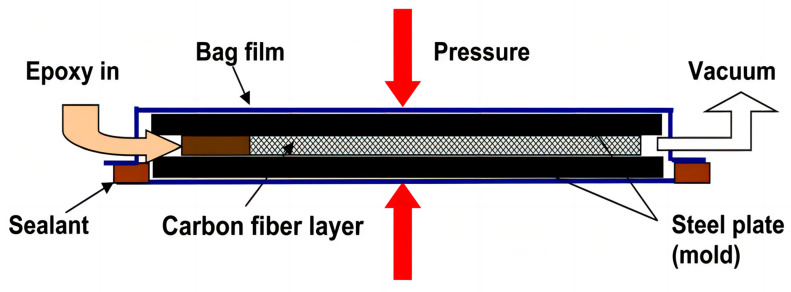
Fabrication of CF composites with vacuum-bagging resin infusion plus a hot press [107].

Yue et al. [107] investigated the effect of oxidation treatment and surface coating on the surface properties of solvated intermediate-phase pitch-based carbon fibers (CFs) to improve the mechanical properties of potentially low-cost CF composites. The CFs were treated with air, O_3,_ CO_2_/H_2_O, or HNO_3_ solution or modified with epoxy resin coating. The treated fiber surfaces were characterized by methylene blue adsorption, NaOH absorption, fragmentation test, composite bending test, optical microscopy and scanning electron microscopy inspection. The flexural strength and modulus of the composites could be improved by using O_3_ oxidized fibers and fiber surface coatings. Optical microscopy and SEM confirmed that composites made with surface-treated fibers had less debonded area than untreated fibers. The CF composites were fabricated using a vacuum-bagging resin infusion technique plus a hot press, and approximately 10 layers of CFs were stacked in the space between the two plates, then packaged in a bagging film and sealed around them with a sealant. The two plastic tubes were mounted on opposite sides of the metal plates [108,109,110]. One was used as the resin-infusion inlet and the other as the vacuum outlet. The bags with CFs inside were placed in a heat press. Different pressures (up to 1.1 MPa) were applied to the CF layer at different stages of the manufacturing process to control the filling density of the CFs, release trapped gases, remove excess resin, and increase the CF content of the resulting composite. The CF composites fabricated with vacuum-sleeved resin infusion plus hot press are shown in Figure 20.

The hydrophobicity of the traditional carbon fiber surface makes it difficult for epoxy resin to infiltrate into the interior of the carbon fiber, and it is largely necessary to impregnate it with a sizing agent [111,112,113,114]. At present, a variety of PEEK-based, PEI-based and other high-temperature-resistant water-based sizing agents have been reported, but in order to improve the dispersion uniformity and stability of the sizing agent, it is usually necessary to add a fixed amount of surfactant during the preparation process. The high molding temperature of carbon fiber composites can easily lead to the pyrolysis of the remaining surfactant on the CF, which results in the generation of interfacial defects and affects the mechanical properties of the carbon fiber composites [115,116].

## 8. Composite-Material Molding

Composite materials are made of two or more substances with different physical and chemical properties, artificially synthesized into a multi-phase solid material [117]. The performance is much better than that of a single material, and, according to the demand for material properties of the material design and manufacturing, it can be made into the desired arbitrary shape of the product, to avoid multiple processing procedures [47,118,119,120,121]. It usually consists of matrix material, a reinforcing material interlayer, and layer structure material; interfacial bonding is the key, including interfacial wetting, dissolution, and a chemical reaction between matrix and reinforcement. Composite material performance characteristics are the following: high specific strength and specific modulus, good fatigue resistance, good high-temperature resistance, good vibration-damping performance, high fracture safety, and good designability. Commonly used reinforcing fibers are the following: carbon fiber, boron fiber, aramid fiber, glass fiber, and silicon carbide fiber whiskers. The thermosetting resins are epoxy, polyimide, etc. The thermoplastic is polyetheretherketone [122,123,124,125,126,127,128,129,130].

Colince et al. [131] prepared a high-strength laminated bamboo composite using rotary-cut bamboo veneer in order to promote the development of the “bamboo instead of steel” program, and which was realized by the hot-press molding method. Orthogonal experiments were carried out on L9 (33), with hot-pressing temperature, pressure, and time as the three influencing factors. Physical properties such as density and moisture content, and mechanical properties such as modulus of rupture (MOR), modulus of elasticity (MOE), shear strength and compressive strength of the samples were tested. From the results of the polar analysis of variance (ANOVA) and analysis of variance (ANOVA), it was observed that higher density and lower moisture content were associated with higher mechanical strength. Within the range of factors selected for testing, a hot-pressing temperature and time of 150 °C and 10 min could contribute to higher density and lower moisture content, and the combination of 150 °C and 50 MPa could produce greater mechanical strength. In the thickness direction, the laminated bamboo composites showed a significant compressive structure.

**Figure 21:**
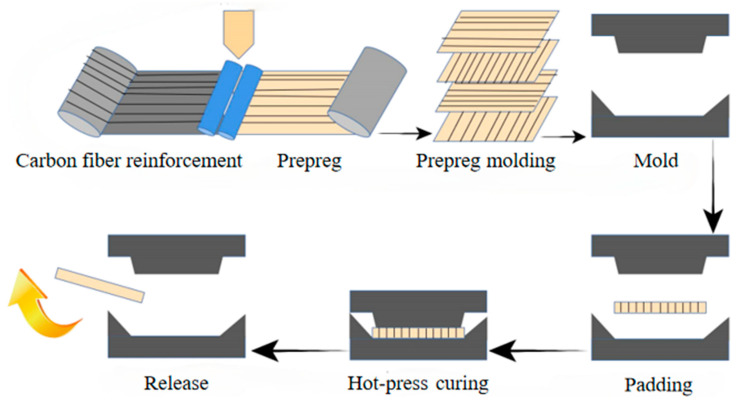
Carbon fiber prepreg compression molding process [75].

The molding process is mostly used for prepreg or fiber braid and resin film composed of laminated materials such as sheet processing and molding; the carbon fiber pretreatment enhances the resin and carbon fiber infiltration, and the carbon fiber is stacked into a metal mold, and then the resin is cast in the carbon fiber, so that it fills the mold cavity. A good time, temperature, pressure, etc., is set up together with hot pressing of the end of the shell molding, and the hot-pressing flowchart is shown in Figure 21. The process is simple, short molding cycle, low energy consumption, low cost, is the most important one of the molding method in industrial production, but the process is suitable for the production of simple structure of the product, for the shape of the complex product processing molding is more difficult [82,132,133,134,135,136,137,138,139].

The carbon-fiber-composite molding process not only has a great influence on the performance of composite structural parts, but also plays a key role in the promotion of carbon-fiber-composite applications [87,140,141,142,143]. The molding process is mostly used for the processing and molding of prepreg or fiber braid and for laminated material composed of resin film, etc. The sheet material cut to the size of the mold is placed in the mold, and the corresponding products can be obtained by heating and pressurizing and then cooling and molding [144,145]. The process is simple, with a short molding cycle, low energy consumption, and low cost, and is the most important type of molding in industrial production; the process is suitable for the production of a simple structure of the product, but the complex shape of the product processing and molding is more difficult [146].

## 9. Conclusions

The pursuit of green and sustainable development has been the basic guideline for all industries in today’s society, due to the expensive price of commercial carbon-fiber composites with bisphenol A-type epoxy resin as the precursor. The process of production and transportation will produce harmful substances, which is not conducive to the concept of sustainable development, and restricts the wide application of carbon fiber composites. Straw tar, as an inexpensive and widely available biomass polymer, is considered as a suitable raw material for carbon fiber composites, due to its high carbon content. The preparation of bio-based epoxy resins using straw tar and lignin is characterized by low cost and high performance, and the surface modification pretreatment of carbon fibers improves their surface bonding. However, the preparation of adhesives from bio-based materials will cause the mechanical properties of carbon fiber sheets to be reduced, and the current adhesives should be developed in the direction of high performance and non-toxicity and harmlessness; the preparation of composites by pressing carbon fibers with synthesized straw-tar-based epoxy resins has a broad market prospect and ecological construction role.

The extraction of phenol derivatives is one of the effective ways of realizing the high value-added utilization of straw tar; the chemical method is mainly based on alkali washing, but the method has the shortcomings of low de-phenolization rate, high acid and alkali dosage, high number of washing times, and serious environmental pollution. Another mainly organic solution is using a solvent to extract phenolic compounds, and the same solvent for the poor selectivity of phenolic compounds, resulting in an unsatisfactory separation effect and other issues.

Due to the unique spatial site resistance and stability of lignin, resulting in a reduction in the activity of chemical reactions, lignin nanosizing effectively overcomes its inherent heterogeneity, poor dispersion, large particle size, and other shortcomings, and provides a new way of thinking for the development of high value-added lignin-based products. Lignin nanoparticles can not only realize the high value-added utilization of lignin, but also solve the safety hazards of the biohazardous nature of traditional nanomaterials. The future development of lignin nanoparticles is expected to focus on the stable preparation and green preparation of particles.

Due to the hydrophobicity of the surface of carbon fiber cloth causing the difficulty of combining the resin with carbon fiber, the addition of a bio-based sizing agent increased the interfacial compatibility between the two. There are many factors affecting the performance of carbon fiber and resin-matrix composites, such as the match between the fiber and resin matrix, quality control in the molding process, parameter optimization, etc. As a reinforcing material for advanced composites, in short, the research on the surface structure and properties of carbon fibers, and the surface modification, will receive more and more attention, and carbon fibers will also play an increasingly important role in many fields.

## Figures and Tables

**Table 1:** Main chemical composition of corn straw tar [22].

Serial Number	Chinese Name	Matching Molecular Formula	Peak Area/%
1	phenol	C_6_H_5_OH	9.57
2	Trans decahydronaphthalene	C_10_H_18_	34.12
3	4-methylphenol	C7H8O	6.51
4	naphthane	C_10_H_18_	17.65
5	2-ethylphenol	C_8_H_10_O	0.46
6	2,4-dimethylphenol	C_8_H_10_O	2.18
7	4-ethylphenol	C_8_H_10_O	12.45
8	2,5-dimethylphenol	C_8_H_10_O	0.57
9	naphthene	C_10_H_8_	1.77
10	2,3-dimethylphenol	C_8_H_10_O	0.64
11	4-isopropyl phenol	C_9_H_12_O	0.46
12	2-propyl phenol	C_9_H_12_O	0.69

## Data Availability

The original contributions presented in the study are included in the article, further inquiries can be directed to the corresponding authors.
